# Research on the influencing factors of the willingness to teach among normal school students: based on Social Cognitive Career Theory

**DOI:** 10.3389/fpsyg.2025.1528336

**Published:** 2025-03-28

**Authors:** Jing Guo, Li Jun Tian

**Affiliations:** ^1^College of Education, Zhejiang Normal University, Jinhua, China; ^2^College of Education, Nanchang Normal University, Nanchang, China

**Keywords:** normal school students, professional identity, learning satisfaction, willingness to teach, Social Cognitive Career Theory, gender

## Abstract

The willingness to teach among normal school students, especially those majoring in primary education, is an important factor affecting the stability of China’s primary education teaching staff and the quality of the training reserve force of primary education teachers. However, currently, a lack of research exists on the willingness to teach among normal school students majoring in primary education from a dynamic theoretical perspective. This study was based on the Social Cognitive Career Theory (SCCT) model. A questionnaire survey method was used to analyze the willingness to teach and its influencing factors among 630 normal school students majoring in primary education. The results indicated a positive correlation between professional identity, learning satisfaction and willingness to teach among normal school students majoring in primary education, of which the professional identity of the normal school students had a significant positive predictive effect on their willingness to teach. Learning satisfaction partially mediated the relationship between the professional identity and willingness to teach of the normal school students; gender played a moderating role in the relationship between the professional identity and willingness to teach of the normal school students. Studying the influence mechanism of willingness to teach from the perspective of SCCT is of great significance in enhancing the willingness of normal school students majoring in primary education and strengthening the stability of teaching.

## 1 Introduction

Attracting and retaining young talent for teaching has always been a prominent concern for policymakers, administrators, and researchers in the field of education. Prior research has demonstrated that, compared with in-service teachers, pre-service teachers are likely to have more enthusiasm for teaching (Klassen and Chiu, 2011), while their willingness to teach tends to change (Day and Gu, 2007; Durksen and Klassen, 2012). Accordingly, various countries have implemented measures to attract more students, especially students who have received professional pre-service education, to choose and enter the teaching industry. Examples of such measures include China’s Free Teacher Education Policy, increasing salary and benefits, and improving the social status of teachers. According to a survey of over 30,000 normal school students in 12 provinces in China, only 58.0% of normal school students consider teaching as their first career choice (Song et al., 2018). Even in high-level normal colleges and universities, which occupy a dominant position in China’s high-quality teacher training system, some students are not primarily seeking to enter the teaching profession (Zhao and Zhu, 2021). Similarly, in Western countries, a certain percentage of quasi-teachers who receive pre-service training do not enter the teaching profession (Goldhaber et al., 2024). The loss of high-quality pre-service teachers is directly related to the quality of teachers. When the school system faces a quantity shortage, it is limited to reducing the qualification requirements of teachers to address the gap in teacher demand, resulting in a decline in teacher quality. To address the issue of teacher turnover among the younger generation, research has shifted its focus to the willingness of normal school students to teach, which refers to the explicit and comprehensive manifestation of their inherent attitude towards the teaching profession during the pre-service education process (Zhang and Lin, 2016). Previous studies have shown that the willingness of normal school students to teach reflects their tendency towards and possibility of pursuing a teaching career in the future. The strength of their willingness to teach directly affects the proportion of them who will engage in teaching and remain in their positions in the future (Song et al., 2018). Therefore, exploring the willingness of normal school students to teach can provide important information to promote their entry and retention, offering insights into research and practice in the selection, training, and professional development of normal school students.

As a subjective tendency, although there is instability and idealization in the willingness to teach, pre-service normal school students’ willingness to teach cannot be ignored as a predictor of their future career choices and long-term commitment to teaching. Previous studies on the factors that influence normal school students’ willingness to teach have primarily focused on the following aspects: First, research on individual factors that affect the willingness of normal school students to teach focuses on the perspective of motivation, which can be summarized via three dimensions: intrinsic motivation, extrinsic motivation, and altruistic motivation (Watt et al., 2012). External motivation describes the economic rewards, job security, and professional reputation that a profession itself brings. Intrinsic motivation addresses the characteristics of teaching per se, including enjoyment of teaching, job satisfaction, and interest in teaching subject(s). Altruistic motivation emphasizes service goals and contributions to society (Heinz, 2015; Kwok et al., 2022). Among the three sources of motivation, compared to external motivation that is easily influenced by external factors and carries the risk of post-employment turnover, intrinsic and altruistic motivations stem from the positive value orientation of individual cognition and are important factors in individuals’ choice of teaching profession, which can better determine their willingness to choose teaching (Flores and Niklasson, 2014; Fokkens-Bruinsma and Canrinus, 2014). Second, starting from school factors, these studies explore the factors that affect the willingness of normal school students to teach, primarily involving the evaluation of teaching quality in the training of normal school students (Horvath et al., 2018; Xu et al., 2022), the recognition of teacher education quality (Song et al., 2018), and the mechanism of impact of various learning supports on the willingness of normal school students to teach (e.g., internship activities, teacher support, and practical communities during the internship process) (Li et al., 2024). Further support has been provided for the existence of a correlation between educational quality and the willingness to teach (Richardson and Watt, 2016). Third, the social impact on normal school students mainly focuses on social and cultural perspectives, including public opinion and gender role culture. In a broader socio-cultural context, normal school students obtain evaluations of the risks associated with teaching (Neugebauer, 2015) and its social reputation (Taimalu et al., 2021) through mass media, social public opinion, and other means, thereby influencing their willingness to teach. Meanwhile, the education industry is one of the most gender-segregated employment sectors in many countries, and this social role culture construction leads to a tendency for female normal school students to choose to work as teachers, negatively affecting the number of men willing to engage in this profession (Lovett, 2014). This is a gender norm replicated on the basis of division of labor (Asimaki and Vergidis, 2013). Overall, previous studies have explored the factors that influence normal school students’ choice to become teachers from different research methods and perspectives. However, further literature analysis reveals that most studies analyze only a single factor, with relatively little research exploring the existence of relationships among various factors, how these relationships drive evolution of the choice to teach, and what mechanisms different factors present, resulting in relatively insufficient explanations for complex interactions. Willingness to teach is not merely a matter of personal cognition; what drives people to teach and remain teaching are complex and is influenced by various individual, contextual, and structural factors (Wu et al., 2025). Therefore, further research should explore the complex factors that affect the willingness of normal school students to teach, as well as the synergistic relationships among multiple factors.

In response to the research gap, this study explores the career choice tendencies of pre-service teachers in normal colleges and universities in Jiangxi Province. Jiangxi Province serves as a useful setting for this inquiry because it has a long history of normal education. Based on the study’s analysis, this paper proposes new solutions to solve the problem of the continuous increase in the teacher turnover rate. Notably, the study is based on Social Cognitive Career Theory (SCCT), which integrates multiple disciplinary perspectives, such as sociology, psychology, and cultural studies. SCCT overcomes the limitations of traditional theories that separate influencing factors and lack dynamics, emphasizing the process and long-term nature of career choice. It not only considers the innate factors of pre-service teacher candidates’ personal characteristics but also the impact of factors such as professional identity and learning satisfaction on their willingness to teach. Additionally, this study notably explores the willingness of normal school students to teach from a dynamic and multidimensional perspective. The study’s concrete evidence is applied to make recommendations on how to strengthen the stability of normal school students’ willingness to teach. This has profound significance for increasing the proportion of normal school students who enter and stay with the teaching profession as well as for the quantity and quality development of teacher education.

## 2 Theoretical basis and research assumptions

### 2.1 Theoretical basis

Lent et al.’s (1994) SCCT, a social cognitive theoretical framework related to careers constructed on the basis of social cognitive theory, focuses on the interactions between individual characteristics, external environmental factors, self-determination and outcome expectations. First, SCCT emphasizes the influence of personal background or personal characteristics, such as gender, domicile and other predisposing factors on career choice; in addition, it highlights that these stable, predisposing antecedents can either negatively or positively affect an individual’s career choice in the form of resistance or support. Second, SCCT emphasizes the interaction between individual and environment. In a social environment with unique characteristics, individuals will gradually accept and internalize social norms, forming a mental structure with social characteristics, which continuously influences their psychological tendencies and behavioral choices towards the external environment. When individuals perceive that the support of the social environment for the goals to be achieved is not ideal or is substantially lower than expected, it is obvious that their career expectations and career choices will deviate from the prescribed track. Third, SCCT emphasizes the significant influence of cognitive regulation on career values and choices. In the process of career development, individuals, as actors with subjective consciousness, can not only improve their adaptation to the social environment through interaction with the objective environment, but also construct their cognition about their careers in the process of information collection and experience acquisition. Consequently, they consciously act to promote the realization of their career goals, which in turn affects the sustainability of their careers.

SCCT regards individual’s career development path as the result of the interaction between multiple career elements, and analyses the individual’s career development process around the relationship between core cognitive variables (self-efficacy, outcome expectations and goals), personal traits (personality psychological tendencies, gender, ethnicity, etc.), and environmental factors (early growth environment, recent environment). The relationship between these elements is used to analyze the process of individual career development, and through the interaction of these elements, to further answer career questions such as how people’s career interests are formed, how people make career choices, how people achieve different levels of career success and stability, and how people experience satisfaction or well-being in the work environment (Lent and Brown, 2013). It not only clarifies the logic of individuals’ current career choices, but also predicts the mechanism of individuals’ career sustainability. Further, it pays attention to the role of individuals’ learning experiences and values on career decision-making and acknowledges the influence of predisposing factors and the social environment (Long et al., 2002), thereby providing an important theoretical basis for the discussion of individuals’ career attitudes and career performance. Therefore, this study relies on Social Cognitive Career Theory as a theoretical framework to investigate the effects of professional identity (cognitive variable), gender (personal trait), and learning satisfaction (domain-specific satisfaction) on the willingness to teach in the pre-service career path development of normal school students.

### 2.2 Impact of normal school students’ professional identity on their willingness to teach

Professional identity is a special form of social identity, in which individuals distinguish themselves from other groups to understand their profession, perceive the value of their profession, and recognize their individual talents (Schein, 1978). The concept of teacher professional identity derives from the exploration of three fundamental questions: what is the “identity” of a teacher, how do others perceive teachers, and what do individuals need to do to become teachers (Rodrigues and Mogarro, 2019). Teachers’ professional identity refers to teachers accepting the teaching profession from the bottom of their hearts and having the ability to positively perceive and evaluate various aspects of it, including cognitive, emotional, volitional, behavioral, and other structural components; hence, they must be willing to engage in the teaching profession for a long time (Beijaard et al., 2000). Since the 1980s, experts and scholars have defined various dimensions of teacher professional identity. Kremer and Hofman (1985) divided it into four dimensions: centripetal, value, solidarity, and self-expression. Whereas, Fang and Mao (2008) divided it into the following four dimensions: professional value, professional emotion, professional ability, and professional social status. Previous studies have shown that in the pre-service stage, the professional identity of normal school students mainly includes: (1) professional emotional identity—individuals’ positive emotions and beliefs related to the teaching profession; (2) professional value identity—individuals’ positive attitude and experience towards the external characteristics of the teaching profession, such as social status, promotion opportunities, and working conditions; (3) professional willingness identity—individuals’ attention to the teaching profession and their tendency to prepare for pre-service work; and (4) professional will identity—individuals’ tendency and will to choose a teaching profession in the future (Zhu and Che, 2021). The formation of professional identity among normal school students is a developmental process, during which a differentiation is made between perception and identification. Normal school students transition from a student identity to a professional one forms a preliminary understanding of the teaching profession (Moussay et al., 2011).

From the perspective of career development stages, normal school students are in the stage of career exploration. Their main task is to develop a career self-concept that reflects their individual career interests and abilities, and to make clear and stable career decisions that are consistent with it. Different from the way in-service teachers obtain career perception and experience, normal school students’ form their career self-concept based more on the professional identity of student status, i.e., their cognitive, emotional, and behavioral experience. Specifically, they relate to their status as normal school students and the teaching profession they are going to engage in on the basis of study, observation, etc. (Wei, 2008), which is a kind of positive attitude related to their career. The Career Interest Model under SCCT (Lent et al., 1994) explores the formation process of individual career interest perception and career choice. Specifically, it describes that as individuals deepen their career learning experience in a certain career field, they exhibit stable career interests in that field and ultimately make corresponding career choices. As a subjective feeling of engaging in a profession, professional identity represents the stability and clarity of an individual’s understanding of their professional talents, interests, and goals (Holland et al., 1993). Combined with existing studies, both professional identity and willingness to teach represent the process of normal school students’ psychological integration of cognitive, emotional, and behavioral tendencies in the pre-service stage. The professional identity of normal school students is the key to their transformation from learners into teachers and the psychological foundation for being a good teacher. Further, these significant endogenous factors affect their willingness to teach (Timoštšuk and Ugaste, 2010). In the career development process of normal school students, individuals’ willingness to teach is based on the establishment of their understanding of the profession; their cognition plays a moderating role between stimulation and behavior. Individuals with higher professional recognition usually have higher academic pursuits and stronger willingness to improve, and are more willing to actively participate in learning related to their future careers (McIlveen et al., 2013), ultimately choosing to work in related professions. At the same time, individuals with higher professional choice intentions usually have a higher degree of recognition of their profession, and hold an optimistic attitude and positive expectations towards career prospects (Rottinghaus et al., 2005). From the perspective of time, normal school students’ professional identity is a value judgement made on the basis of career cognition, which occurs in the middle and early stages of attitude development; their willingness to teach forms a stable emotional connection on the basis of professional identity, which stabilizes in the middle and late stages of attitude development (Wang et al., 2010). Thus, the professional identity of normal school students is not only the foundation for the development of their willingness to teach, but also an important factor affecting their willingness to teach. Therefore, this study proposes Hypothesis H1: The professional identity of normal school students can positively influence their willingness to teach.

### 2.3 The mediating effect of learning satisfaction

The Work and Life Satisfaction Model of SCCT integrates the relationship between individuals and their environment (Kim et al., 2016). Its basic viewpoint contends that emotions and satisfaction can extend to a person’s non-work time in specific areas, such as the work environment (Judge and Ilies, 2004), having a certain influence in the development of their career course. Learning satisfaction is formed in the specific context of students’ education, which is a subjective experience that objectively exists in the pre-service education stage, describing a positive psychological state. Currently, a unified standard for the concept of learning satisfaction does not exist. Some scholars understand this concept from the perspective of psychological processes, defining learning satisfaction as learners’ psychological feelings towards the realization of their needs or desires in teaching activities, interpersonal relationships, environmental facilities, and other aspects of learning and life (Shu and Xu, 2014). Some scholars (Demir and Mukhlis, 2017) have defined learning satisfaction as a short-term attitude of students after their educational experience, based on the perceived gap between expectations and experiences. The concept used in this study focuses on comprehensively assessing students’ satisfaction from three aspects: teachers, students, and school environment. Learning satisfaction is quantified as measuring students’ overall perception and final evaluation of the learning environment, teaching management, teacher teaching, and interpersonal relationships during the learning process (Wen, 2015). Previous studies have found that learning satisfaction can significantly affect the willingness to teach, and according to Darling-Hammond et al. (2002), a significant positive correlation exists between normal school students’ judgement of the quality of teacher education and their subjective willingness to teach. In a survey of 198 normal school students, Bruinsma and Jansen (2010) found that pre-service teachers who had a positive experience with the quality of pre-service education had a stronger motivation and willingness to choose the teaching profession, and stayed in the teaching profession for a longer period of time. However, professional identity is also affected by normal school students’ learning satisfaction. Stufflebeam et al. (2000) argue that during the implementation of normal education, normal school students’ satisfaction with factors such as curricular support provided on the school side and pedagogical guidance on the teachers’ side is one of the main factors affecting their professional identity.

Previous studies have identified a certain correlation between professional identity, willingness to teach, and learning satisfaction: normal students gain initial knowledge of the teaching profession at the stage of pre-service education, and as their satisfaction with the professional learning process increases, their acceptance and recognition of the major they are studying enhances, which in turn has a positive impact on their future career positioning and choices, helping them establish a rational understanding of their willingness to teach. Therefore, this study proposes Hypothesis H2: Learning satisfaction plays a mediating role in the professional identity and willingness to teach among normal school students.

### 2.4 The regulatory role of gender

SCCT considers gender an important influencing factor, mainly based on the research of Hackett and Betz (1981), which found that individuals in non-traditional gender occupational fields have lower career interests, while individuals in traditional gender occupational fields have higher career interests. The socialization of gender roles has a significant impact on career choice behavior. Tokar et al. (2007) showed that the socialization process of individual gender roles prompts individuals to selectively participate in career activities that are consistent with their gender cognition, while avoiding other types of activities, resulting in a tendency and concentration of career learning experience and career choices for individuals of different genders. Therefore, this study proposes Hypothesis H3: Gender plays a moderating role in the prediction of professional identity and willingness to teach.

In summary, this study takes normal school students as the research object and intends to study the mediating role of learning satisfaction and the moderating role of gender in the relationship between professional identity and willingness to teach, with a view to understand the influence of normal school students’ willingness to teach and its mechanism of action.

## 3 Materials and methods

### 3.1 Study participants

Normal colleges and universities are the main bases for training primary education teachers in China, and normal school students majoring in primary education are the main source of primary education teachers. Their firm willingness to teach as primary school teachers has become a core issue of concern in China’s pre-service education training stage. Normal colleges and universities in Jiangxi Province, with their long history and unique cultural background gradually developed hierarchical and distinctive patterns and became important educational bases in East China. Given the richness of this setting for the topic, this study used convenience sampling to select 650 normal school students majoring in primary education from normal colleges and universities in Jiangxi Province as the research sample. Among these students are 148 male students (23.5%) and 482 female students (76.5%). The distribution of data also reflects that the population choosing primary education majors in China is mainly female.

### 3.2 Ethical issues

Before commencement, the study was reviewed by a committee of graduate school of university and obtained the ethical approval. All participants voluntarily participate in the study based on informed consent. The researcher explains the research purpose, process, potential risks and benefits to the participants in written and oral form, and promises that the data will only be used for academic purposes. During the data collection process, all questionnaires are anonymous and encrypted storage and access control measures are taken to ensure data security and prevent any individual privacy leakage or psychological harm due to research.

### 3.3 Research tools

#### 3.3.1 Professional Identity Scale for Normal School Students

The Professional Identity Scale for Normal School Students was adapted from the Professional Identity Scale for Publicly-funded Normal School Students compiled by Zhu and Che (2021), with a total of 16 questions including four dimensions, namely, professional emotional identity, professional value identity, professional willingness to identify, and professional will to identify. The questionnaire was scored on a five-point Likert scale, with 1–5 indicating “very non-conformative” to “very conformative,” and one of the negative questions was assigned a reverse value, with higher scores representing higher professional identity. The overall Cronbach’s *α* coefficient of the scale in this study was 0.921, and the Cronbach’s α coefficients of the four dimensions were 0.948, 0.960, 0.921, and 0.892, respectively. The results of the validated factor analyses showed that the desirable fit indices were χ^2^ /df = 2.55, GFI = 0.94, IFI = 0.95, TLI = 0.91, CFI = 0.92, CFI = 0.95, and TLI = 0.91. Additionally, CFI = 0.92, and RMSEA = 0.05, indicating good reliability and validity of the scale.

#### 3.3.2 Willingness to Teach Scale

The Willingness to Teach Scale was adapted from the Pre-school Education Students’ Intention to Teach Scale by Geng (2020), which consists of 15 questions, including four dimensions—perception of teaching, environment of teaching, treatment of teaching, and intention to teach. The questionnaire was scored on a five-point Likert scale, with 1–5 indicating “very non-compliant” to “very compliant,” and the higher the score, the higher the intention to teach. The overall Cronbach’s *α* coefficient of the scale in this study was 0.932, and the Cronbach’s α coefficients of the four dimensions were 0.955, 0.919, 0.921, and 0.852, respectively. The results of the validated factor analyses showed that the desirable fit indices were χ2 /df = 2.64, GFI = 0.93, IFI = 0.96, TLI = 0.93, CFI = 0.92, CFI = 0.95, and TLI = 0.95. CFI = 0.92, and RMSEA = 0.05, indicating good reliability and validity of the scale.

#### 3.3.3 Learning Satisfaction Scale

The Learning Satisfaction Scale was adapted from the College Student Learning Satisfaction Survey Questionnaire developed by Wen (2018), which consists of 21 questions, including four dimensions—learning environment, teaching management, teacher teaching, and interpersonal relationship. The questionnaire was scored on a five-point Likert scale, with 1–5 indicating “very dissatisfied” to “very satisfied,” with higher scores indicating higher learning satisfaction. The overall Cronbach’s α coefficient of the scale in this study was 0.932, and the Cronbach’s α coefficients of the four dimensions were 0.972, 0.959, 0.932, and 0.951, respectively. Validated factor analyses showed that the desirable fit indices were χ^2^ /df = 2.24, GFI = 0.92, IFI = 0.92, TLI = 0.96, CFI = 0.93, and TLI = 0.96, respectively. Furthermore, CFI = 0.93, and RMSEA = 0.04, indicating good reliability and validity of the scale.

### 3.4 Research program

This study used a questionnaire survey method. To improve the reliability and accuracy of data acquisition and minimize the distortion of questionnaire data caused by respondents not answering carefully, one trap question and one negative scoring question were included in the questionnaire to screen the data.

The questionnaires were distributed on-site at the research school with the consent of the relevant teachers in charge at the normal schools. Before the questionnaire was administered, we introduced students to its purpose and requirements, and explained the voluntary and confidential principles of the test. We also provided on-site answers to any questions that students had while completing the questionnaire.

After collecting the questionnaire, we organized and screened it. First, we conducted a preliminary manual screening of the questionnaire; subsequently, we screened the data using statistical software to eliminate invalid questionnaires. Questionnaires were deemed invalid based on the following criteria: presenting regular responses to answer options; more than 10% of the questions in the questionnaire were missed; more than 90% of the questions had the same answer; a clear contradiction between the tendency to answer reverse questions and the tendency to answer forward questions; wrong answers to trap questions. Despite taking various measures to improve the validity of the data, 20 questionnaires were found to be invalid and were accordingly excluded from the sample. In total, 630 valid questionnaires were obtained, with an effective rate of 96.9%.

### 3.5 Data processing

SPSS 26.0 and PROCESS were used for data analysis. First, to ensure that this study had no serious common methodological bias due to the use of self-reported methodology, a common methodological bias test was conducted using the Harman one-way test. Subsequently, SPSS 26.0 was used to initially collate the collected data for descriptive statistics as well as correlation analyses among the variables. Finally, we used the SPSS macro program PROCESS to conduct mediation and moderation effect tests.

## 4 Results

### 4.1 Common method deviation test

Due to the use of self-reporting by participants, common method bias may exist. Therefore, emphasis was placed on the confidentiality and anonymity of the questionnaire during the actual administration and it was stated that the data were restricted to scientific research use to try to control the source of common method bias. After recovering the data, a common method deviation test was conducted using the Harman one-way test (Podsakoff et al., 2003). The results showed that 10 factors with eigenvalues greater than 1 were obtained without rotation, and the amount of variation explained by the first factor was 38.65%, which met the statistically required criterion of less than 40%, i.e., the data in this study did not suffer from a serious problem of common method bias.

### 4.2 Descriptive statistics and differential statistical analysis

This study conducted a descriptive statistics and demographic variable differences analysis on professional identity, learning satisfaction, and willingness to teach among normal school students majoring in primary education. The findings are shown in Table 1. The professional identity score of normal school students majoring in primary education is 3.10, which is greater than the theoretical median value of 3 and is in the upper middle level; the learning satisfaction score is 3.39, which is greater than the theoretical median value of 3 and is in the upper middle level; and the willingness to teach score is 2.69 and is in the lower middle level.

**Table 1:** Descriptive statistics and analysis of differences in demographic variables (*n* = 630).

Demographic variables		Professional identity	Learning satisfaction	Willingness to teach
	M ± SD	3.10 ± 0.86	3.39 ± 0.72	2.69 ± 0.88
Grade	Freshman 1	3.92 ± 0.6	3.91 ± 0.65	3.68 ± 0.39
	Sophomore 2	3.28 ± 0.38	3.55 ± 0.41	2.95 ± 0.11
	Junior 3	2.98 ± 0.4	3.37 ± 0.49	2.49 ± 0.19
	Senior 4	2.2 ± 0.62	2.77 ± 0.57	1.62 ± 0.38
	*F*	334.809^**^	135.892^**^	1377.167^**^
	LSD	1 > 2, 1 > 3, 1 > 4. 2 > 3, 2 > 4, 3 > 4	1 > 2, 1 > 3, 1 > 4. 2 > 3, 2 > 4, 3 > 4	1 > 2, 1 > 3, 1 > 4. 2 > 3, 2 > 4, 3 > 4
	η^2^	0.616	0.394	0.868
Path to further education	College Entrance Examination	2.7 ± 0.68	3.14 ± 0.62	2.19 ± 0.60
	Upgrading Examination	3.88 ± 0.61	3.89 ± 0.63	3.64 ± 0.40
	*T*	14.219^**^	14.337^**^	31.665^**^
	Cohen’s d	1.80	1.20	2.68
Frequency of participation in practice	Participated in two practices 1	3.29 ± 0.5	3.57 ± 0.51	2.94 ± 0.32
	Participated in one practice 2	2.73 ± 0.33	3.18 ± 0.46	2.14 ± 0.12
	Participated in more than two practices 3	4.03 ± 0.68	4.18 ± 0.52	3.96 ± 0.31
	Not involved in practice 4	2.64 ± 0.57	2.02 ± 0.63	1.45 ± 0.31
	*F*	145.225^**^	387.075^**^	1650.065^**^
	LSD	1 > 2, 1 < 3, 1 > 4. 2 < 3, 2 > 4, 3 > 4	1 > 2, 1 < 3, 1 > 4. 2 < 3, 2 > 4, 3 > 4	1 > 2, 1 < 3, 1 > 4. 2 < 3, 2 > 4, 3 > 4
	η^2^	0.420	0.247	0.615

* *p* < 0.05, ** *p* < 0.01, *** *p* < 0.001.

The differential analysis of professional identity, learning satisfaction, and willingness to teach among normal school students majoring in primary education was based on three demographic variables: grade level, path to further education, and frequency of participation in practice. Simultaneously, we calculated the effect value. The results of the analysis are reported below.

The study used independent sample *t*-tests to examine the differences in professional identity, learning satisfaction, and willingness to teach among normal school students majoring in primary education with different paths to further education. We calculated Cohen’s d to determine the magnitude of the effect of the differences between the two groups. A Cohen’s d less than 0.2 indicates minimal differential effects, a Cohen’s d value between 0.2 and 0.5 indicates that the differential effect is small, a Cohen’s d between 0.5 and 0.8 indicates a moderate differential effect, and a Cohen’s d is greater than 0.8 indicates a significant differential effect (Cohen, 1988). The results showed significant differences in professional identity (*T* = 14.219, *p* < 0.01), learning satisfaction (*T* = 14.337, *p* < 0.01), and willingness to teach (*T* = 31.665, *p* < 0.01) among normal school students majoring in primary education with different paths to further education. Notably, students who entered normal schools with Upgrading Examinations had higher mean values in terms of profession identity, study satisfaction, and willingness to teach than students who entered by National College Entrance Examination (the differential effect values were 1.80, 1.20, and 2.68, respectively). These findings indicate that there are significant differences in the effects of professional identity, learning satisfaction, and willingness to teach.

The study used one-way analysis of variance to examine differences in professional identity, learning satisfaction, and willingness to teach among normal school students majoring in primary education with different grades and frequencies of participation in practice, calculating the effect size as η^2^. An η^2^ less than 0.01 indicates the absence of differential effects, an η^2^ between 0.01 and 0.06 indicates the presence of a small differential effect, an η^2^ between 0.06 and 0.14 indicates the presence of moderate differential effects, and an η^2^ greater than 0.14, indicates significant differential effects (Cohen, 1988). The results showed significant differences in terms of professional identity (*F* = 334.809, *p* < 0.01), learning satisfaction (*F* = 135.892, *p* < 0.01), and willingness to teach (*F* = 1377.167, *p* < 0.01) among normal students majoring in different grades of primary education. A multiple comparison analysis showed that students in the first-year stage had the highest professional identity, learning satisfaction, and willingness to teach, and students in the fourth-year stage had the lowest professional identity, learning satisfaction, and willingness to teach. Broadly, professional identity, learning satisfaction, and willingness to teach decreased with increases in the grade level of specialization. The differential effect values for professional identity, learning satisfaction, and willingness to teach were all significant at 0.616, 0.394, and 0.868.

Similarly, significant differences were observed in professional identity (*F* = 145.225, *p* < 0.01), learning satisfaction (*F* = 387.075, *p* < 0.01), and willingness to teach (*F* = 1650.065, *p* < 0.01) among normal school students majoring in primary education with different frequencies of participation in practice. The analysis of multiple comparisons showed that students who had participated in more than two practices had the highest scores for professional identity, learning satisfaction, and willingness to teach, while students who had not participated in practices had the lowest scores for all three scales. Broadly, professional identity, learning satisfaction, and willingness to teach exhibited a decreasing trend with the number of times they participate in practice. The differential effect values for professional identity, learning satisfaction, and willingness to teach were all significant at 0.420, 0.247, and 0.615, respectively.

### 4.3 Correlation analysis between professional identity, learning satisfaction, and willingness to teach

The Pearson correlation test was used to test the correlation of the three scales of learning satisfaction, professional identity, and willingness to teach as a whole. As shown in Table 2, the correlation coefficients of learning satisfaction with professional identity and willingness to teach were 0.719 and 0.685, respectively. The correlation coefficient of professional identity and willingness to teach was 0.862, and the coefficients have passed the significance test with a significance level of 1%, which indicates that there was a significant positive correlation between learning satisfaction, professional identity, and willingness to teach.

**Table 2:** Analysis of overall correlation between professional identity, learning satisfaction, and willingness to teach (*n* = 630).

	Learning satisfaction	Professional identity	Willingness to teach
Learning satisfaction	1		
Professional identity	0.719^**^	1	
Willingness to teach	0.685^**^	0.862^**^	1

* *p* < 0.05, ** *p* < 0.01, *** *p* < 0.001.

Using the Pearson correlation test, we conducted correlation analysis on the specific dimensions of professional identity, learning satisfaction, and willingness to teach. As shown in Table 3, a significant positive correlation exists between the dimensions of learning satisfaction, professional identity, and willingness to teach. Therefore, it is necessary to use the data provided by the Pearson correlation matrix to explore the relationships between the variables in depth.

**Table 3:** Correlation analysis of dimensions of professional identity, learning satisfaction, and willingness to teach (*n* = 630).

	1	2	3	4	5	6	7	8	9	10	11	12
1.Learning environment	1											
2.Teaching management	0.298^**^	1										
3.Teacher teaching	0.294^**^	0.292^**^	1									
4.nterpersonal relationship	0.309^**^	0.268^**^	0.254^**^	1								
5.Emotional identification	0.513^**^	0.329^**^	0.190^**^	0.216^**^	1							
6.Value identification	0.522^**^	0.427^**^	0.306^**^	0.270^**^	0.223^**^	1						
7.Willingness identification	0.471^**^	0.349^**^	0.230^**^	0.192^**^	0.381^**^	0.323^**^	1					
8.Will identification	0.467^**^	0.384^**^	0.228^**^	0.229^**^	0.372^**^	0.635^**^	0.295^**^	1				
9.Perceptions of teaching	0.645^**^	0.385^**^	0.237^**^	0.286^**^	0.625^**^	0.582^**^	0.519^**^	0.573^**^	1			
10.Environment of teaching	0.636^**^	0.397^**^	0.231^**^	0.251^**^	0.584^**^	0.576^**^	0.550^**^	0.528^**^	0.685^**^	1		
11.Treatment of teaching	0.409^**^	0.240^**^	0.113^**^	0.211^**^	0.398^**^	0.367^**^	0.315^**^	0.294^**^	0.469^**^	0.444^**^	1	
12.Intention to teach	0.395^**^	0.215^**^	0.280^**^	0.170^**^	0.412^**^	0.315^**^	0.341^**^	0.261^**^	0.422^**^	0.393^**^	0.171^**^	1

* *p* < 0.05, ** *p* < 0.01, *** *p* < 0.001.

### 4.4 Analysis of the mediating effect of learning satisfaction

With professional identity as the independent variable, learning satisfaction as the mediator variable, and willingness to teach as the dependent variable, the mediation effects were tested using the bias-corrected percentile Bootstrap method using Model 4 of the SPSS macro program PROCESS developed by Hayes (2013), controlling for gender, grade, path to further education, and frequency of participation in practice.

First, we conducted regression analyses with professional identity as the independent variable and willingness to teach as the dependent variable. The results showed that professional identity significantly and positively predicted the willingness to teach of normal school students majoring in primary education (*β* = 0.86, *t* = 42.54, *p* < 0.001). Subsequently, the mediating effect of learning satisfaction between professional identity and the willingness to teach of normal school students majoring in primary education was tested. The results showed that when both professional identity and learning satisfaction were put into the regression equation, professional identity had a significant direct predictive effect on the willingness to teach of normal school students majoring in primary education (*β* = 0.32, *t* = 14.70, *p* < 0.001), learning satisfaction had a significant direct predictive effect on the willingness to teach of normal school students majoring in primary education (*β* = 0.06, *t* = 3.26, *p* < 0.01), and the mediating effect size of the bootstrap confidence interval did not contain 0 ([0.02, 0.09]). Therefore, the partial mediating effect of learning satisfaction on professional identity and normal school students’ willingness to teach in primary education is significant (see Table 4).

**Table 4:** Regression analyses of the mediating role of learning satisfaction between professional identity and willingness to teach.

Regression equation (*N* = 630)		Overall fit index			Significance of regression coefficients			
Outcome variable	Predictor variable	*R*	*R* ^2^	*F*	*β*	Bootstrap lower limit	Bootstrap upper limit	*t*
Learning satisfaction	Grade	0.73	0.53	142.87^***^	−0.07	−0.19	0.05	−1.10
	Gender				0.14	−0.01	0.27	1.96
	Path to further education				0.05	−0.21	0.30	0.36
	Frequency of participation in practice				−0.07	−0.13	−0.01	−2.35^*^
	Professional identity				0.59	0.51	0.68	13.60^***^
Willingness to teach	Grade	0.96	0.91	1087.56^***^	−0.49	−0.54	−0.44	−18.28^***^
	Gender				0.06	0.00	0.12	2.00^*^
	Path to further education				0.08	−0.03	0.20	1.50
	Frequency of participation in practice				−0.02	−0.05	0.00	−1.66
	Professional identity				0.32	0.28	0.36	14.70^***^
	Learning satisfaction				0.06	0.02	0.09	3.26^**^

* *p* < 0.05, ** *p* < 0.01, *** *p* < 0.001. Variables in the model were standardized and then brought into the regression equation.

**Figure 1:**
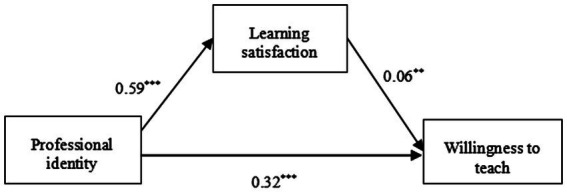
Path coefficient diagram of professional identity, learning satisfaction, and the willingness to teach.

According to the analysis of the mediating effect, the specific path coefficient of the mediating effect of learning satisfaction on the relationship between professional identity and the willingness to teach of normal school students majoring in primary education is illustrated in Figure 1.

The bootstrap test (see Table 5) showed that the total effect was 0.36, with a 95% CI of [0.32, 0.39]; the direct effect was 0.32, with a 95% CI of [0.28, 0.36]; and the mediating effect of learning satisfaction was 0.04, with a 95% CI of [0.01, 0.06], which accounted for 11.11% of the total effect. This suggests that professional identity can have both a direct effect on willingness to teach and a mediating effect through the variable of learning satisfaction; that is, learning satisfaction plays a partial mediating role in the relationship between professional identity and willingness to teach.

**Table 5:** Subdivision table of total effect, direct effect, and mediating effect.

Project	Effect	SE	LLCI	ULCI	Effect size
Total effect	0.35	0.02	0.31	0.39	
Direct effect	0.32	0.02	0.27	0.36	88.89%
Mediating effect	0.03	0.01	0.01	0.06	11.11%

### 4.5 Moderating effect test for gender

First, the independent variable of professional identity was centralized. Then, with professional identity as the independent variable, gender as the moderating variable, and willingness to teach as the dependent variable, a moderated effects test was conducted using Model1 of the SPSS macro program PROCESS, controlling for grade, path to further education, and frequency of participation in practice. The results indicated that professional identity had a significant direct predictive effect on normal school students’ willingness to teach in primary education (*β* = 0.42, *t* = 15.09, *p* < 0.001), gender had a non-significant direct predictive effect on normal school students’ willingness to teach in primary education (*β* = 0.28, *t* = 1.01, *p* > 0.05), and the interaction between professional identity and gender had a significant direct predictive effect on normal school students’ willingness to teach in primary education (*β* = 0.28, *t* = 1.01, *p* > 0.05). Intention to teach was significant (*β* = −0.10, *t* = −3.29, *p* < 0.01), indicating that gender had a significant moderating effect on professional identity and willingness to teach among normal school students majoring in primary education (see Table 6).

**Table 6:** Regression analysis of the moderating effect of gender on professional identity and willingness to teach.

Regression equation (*N* = 630)		Overall fit index			Significance of regression coefficients			
Outcome variable	Predictor variable	*R*	*R* ^2^	*F*	*β*	Bootstrap lower limit	Bootstrap upper limit	*t*
Willingness to teach	Grade	0.96	0.91	1087.95^***^	−0.44	−0.49	−0.39	−18.68^***^
	Path to further education				0.08	−0.02	0.18	1.62
	Frequency of participation in practice				−0.01	−0.04	0.01	−1.20
	Professional identity				0.42	0.37	0.48	15.09^***^
	gender				0.28	−0.03	0.08	1.01
	Professional identity × gender				−0.10	−0.16	−0.04	−3.29^**^

* *p* < 0.05, ** *p* < 0.01, *** *p* < 0.001. Independent variables in the model are centered and then brought into the regression equation.

**Figure 2:**
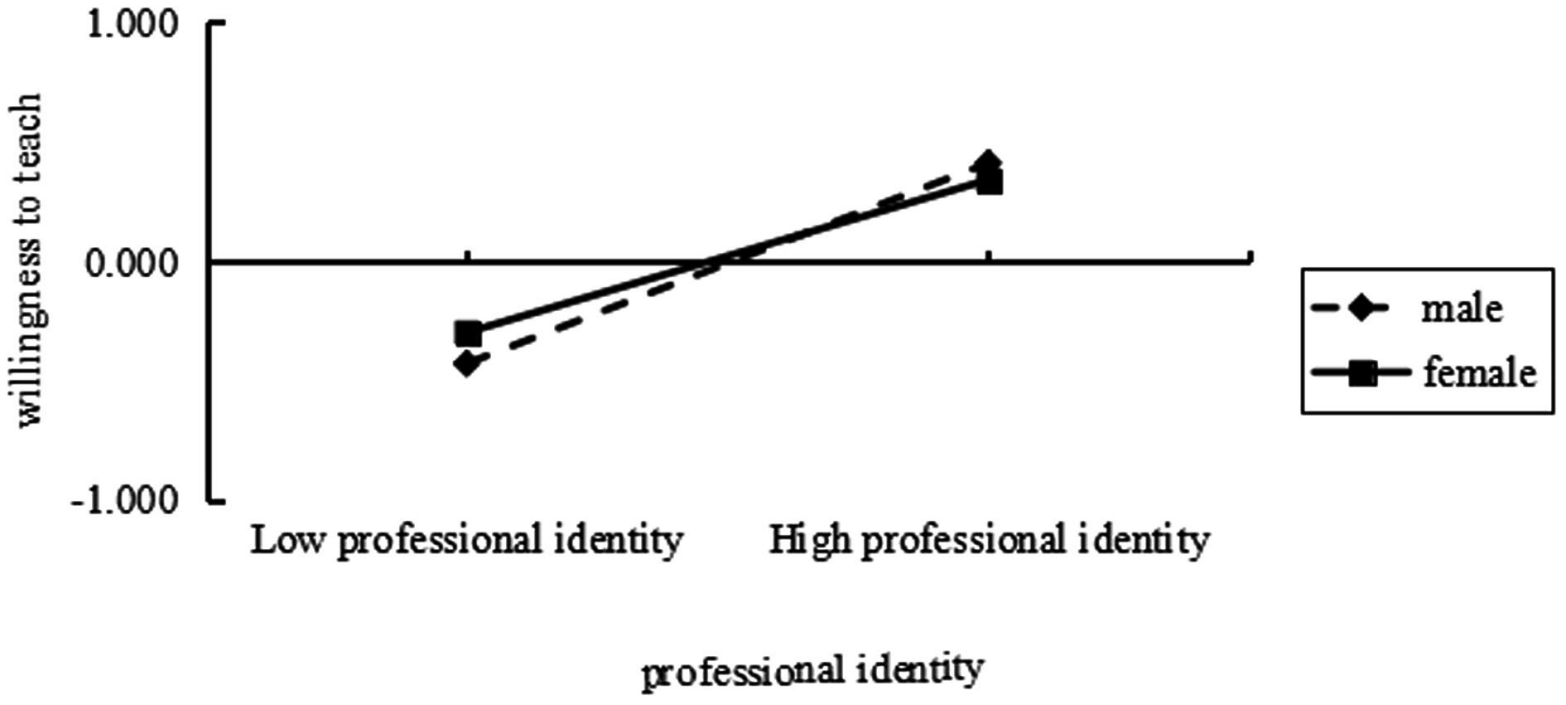
The moderating role of gender on the relationship between professional identity and the willingness to teach among normal school students majoring in primary education.

To further test the moderation effect, simple slope plots were drawn for different genders (male = 0, female = 1) (Figure 2). For male, professional identity had a significant effect on willingness to teach (*β* = 0.48, *p* < 0.001, 95% CI [0.42, 0.54]), and for female, professional identity also had a significant effect on willingness to teach (*β* = 0.37, *p* < 0.001, 95% CI [0.32, 0.42]), with a difference of 0.11 in the level of the two. This indicates that the higher the professional identity of individual male students, the higher the level of willingness to teach compared to female students.

## 5 Discussion

### 5.1 Professional identity positively influences willingness to teach

The results of this study show that the professional identity of normal school students majoring in primary education significantly and positively affects their willingness to teach. This means that when normal school students’ professional identity becomes more stable, their willingness to teach remains strong even when they face various inconveniences and setbacks. This conclusion is consistent with the findings of many previous studies (Hu and Huang, 2016; Johnson and Birkeland, 2016). It also supports the basic viewpoint of SCCT that emphasizes the importance of cognitive regulation on career choice (Long et al., 2002). Social cognitive occupational theory also holds that individual cognition determines a person’s behavioral choices, and the concrete expected outcomes affect individuals’ overall occupational choices (Betz and Hackett, 2006). For the group of normal school students, the educational experience and accumulation gained from professional learning and educational practice construct their understanding of the teaching profession, which directly affects their professional identity. They classify themselves as part of the teaching community and develop a sense of belonging to it. The higher the identification and approval of normal school students towards the value and significance of the teaching profession and their future career, the firmer their inclination towards teaching, thereby further affecting the stability of individual teaching behavior choices.

### 5.2 Learning satisfaction mediates professional identity and willingness to teach

The results of this study indicate that learning satisfaction plays a mediating role between normal school students’ professional identity and their willingness to teach. SCCT suggests that cognition and experience function as a memory cue that influences an individual’s assessment of future behavioral events as well as the implementation of decision-making (O’Neil, 1981). Initially, normal school students majoring in elementary education have an understanding and love of the basic education career at the time of their professional choices. Gradually, they deepen their understanding and experience of their major through their pre-service school life and growth practice, forming unique educational memories that satisfy their desires and needs during learning activities. These memory clues inform their cognition and choice of teaching profession in the form of learning satisfaction. In previous studies, learning satisfaction has been regarded as a process variable closely related to the professional development of normal school students. The theoretical model of normal school students’ professional identity development proposed by Beauchamp and Thomas (2006) suggests that normal school students form their student identity of the teaching profession from their learning experiences. Then, through satisfaction with two important intervention points—theoretical learning and teaching practice—they achieve the transformation from student identity to professional identity, and ultimately choose their career. Darling-Hammond et al. (2002) study also indicated that normal school students accept the teaching profession earnestly and develop positive perceptions and evaluations of various aspects of the teacher education process, which affects their willingness to teach and whether they can adhere to it. Overall, normal school students in primary education who are satisfied with the learning process at the pre-service stage of education will further deepen their educational memory, helping them to realize the transition from student identity to professional identity in their learning experience. This, in turn, positively affects their willingness to choose the teaching profession.

### 5.3 The moderating role of gender

In this study, gender played a moderating role in the relationship between normal school students’ professional identities and their willingness to teach in primary education. The results for males had higher significance levels than those of females. For a long time, the profession’s emphasis on maternal qualities, such as love, patience, tolerance, enthusiasm, affinity, and sympathy, caused the profession to be perceived as one meant for women. This created occupational gender segregation in the field of primary education, which gave rise to the feminization of the primary school teaching force (Murray, 1996; Gao and Yu, 2018). Multiple specialized studies—both within China and beyond—have shown that while teaching remains gendered and teachers implicitly implement gender education, these differences do not affect the quality of teachers’ teaching. Nevertheless, the gender concept in the primary school workplace needs to be improved (Sabbe and Aelterman, 2007; Li, 2024).

Within the Chinese institutional framework, teaching with bianzhi is considered an in-system sector career that is an “iron rice bowl” (Chung and Huang, 2012; Ye et al., 2021). In other words, state administrative staff appear to have relatively high social status. This type of citizenship and the teaching profession greatly appeals to Chinese young people for its career security and welfare system (Chan, 2017). Accordingly, lifetime stable jobs, government allocated salaries, public housing subsidies and reliable medical insurance have gradually made men satisfied with the security brought by the profession of primary school teachers, encouraging them to join the profession. Existing research shows that the glass escalator effect in the teaching profession exists in both the recruitment stage and the professional development stage (Williams, 1992). The “female profession” has a preference for men in recruitment, and the recruitment system is moderately biased towards men. As a result, the career development space for male future teachers is better than that for females. This also leads to a higher sense of professional identity among men towards teachers, resulting in a stronger willingness to teach.

## 6 Conclusion

This study explored the relationship between three factors affecting the willingness to teach of normal school students majoring in elementary education and examined the mechanisms of professional identity, learning satisfaction, and gender on the willingness to teach. The study shows that: (1) The professional identity, learning satisfaction, and willingness to teach of normal school students majoring in elementary education are correlated pairwise, with professional identity significantly predicting the willingness to teach; (2) the mediating effect of learning satisfaction is significant between professional identity and the willingness to teach; and (3) the moderating effect of gender is significant between professional identity and the willingness to teach.

These findings suggest that the willingness of normal school students to teach is not only influenced by external factors such as family, society, and teacher professional treatment, but also by their satisfaction with school education, identification with the teaching profession, and gender differences in the profession. Therefore, in the pre-employment stage of cultivating the willingness of normal school students, gender differences should be considered and attention should be paid to normal school students’ professional identity and learning satisfaction.

## 7 Limitations and future research

There are still some shortcomings in this study. Firstly, due to limited manpower, the sampling size of this study was limited to local normal colleges and universities in Jiangxi Province, which limits the generalizability of the conclusions. Secondly, this study focused on normal school students majoring in primary education, and the sampling was not diverse. Thirdly, due to the comprehensive influence of various internal and external factors on willingness to teach, this study only considered three factors—gender, learning satisfaction, and professional identity—and thus had a limited breadth.

In response to the limitations of the above research, future studies should consider the following. Firstly, the setting should be expanded to other provinces to conduct more extensive empirical research. Secondly, the sample types should be enriched to further explore whether there are differences in the influence mechanism of teaching willingness between normal school students majoring in primary education and other categories of normal school students (e.g., directional trained normal school students, free-charge normal school students). Thirdly, the scope of the research should be expanded to investigate other variables affecting willingness to teach.

## Data Availability

The raw data supporting the conclusions of this article will be made available by the authors, without undue reservation.
